# Diabetes Mellitus Induces Alzheimer’s Disease Pathology: Histopathological Evidence from Animal Models

**DOI:** 10.3390/ijms17040503

**Published:** 2016-04-05

**Authors:** Nobuyuki Kimura

**Affiliations:** Section of Cell Biology and Pathology, Department of Alzheimer’s Disease Research, Center for Development of Advanced Medicine for Dementia, National Center for Geriatrics and Gerontology (NCGG), Gengo 35, Moriika, Obu, Aichi 474-8511, Japan; kimura@ncgg.go.jp; Tel.: +81-562-44-5651 (ext. 6404); Fax: +81-562-46-8569

**Keywords:** Alzheimer’s disease, animal model, diabetes mellitus, insulin resistance, pathology

## Abstract

Alzheimer’s disease (AD) is the major causative disease of dementia and is characterized pathologically by the accumulation of senile plaques (SPs) and neurofibrillary tangles (NFTs) in the brain. Although genetic studies show that β-amyloid protein (Aβ), the major component of SPs, is the key factor underlying AD pathogenesis, it remains unclear why advanced age often leads to AD. Interestingly, several epidemiological and clinical studies show that type II diabetes mellitus (DM) patients are more likely to exhibit increased susceptibility to AD. Moreover, growing evidence suggests that there are several connections between the neuropathology that underlies AD and DM, and there is evidence that the experimental induction of DM can cause cognitive dysfunction, even in rodent animal models. This mini-review summarizes histopathological evidence that DM induces AD pathology in animal models and discusses the possibility that aberrant insulin signaling is a key factor in the induction of AD pathology.

## 1. Introduction

Alzheimer’s disease (AD), the most common cause of dementia, is a progressive and fatal neurodegenerative disorder in which certain types of neurons in particular brain regions degenerate, resulting in severe neuronal loss. AD is characterized by two major pathological hallmarks: senile plaques (SPs) and neurofibrillary tangles (NFTs) [1,2,3]. This advancing pathology is believed to underlie the clinical presentation of memory deficiency first, followed by the steady loss of judgement, verbal fluency, reasoning skills, and other cognitive functions. SPs are the extracellular deposition of aggregated β-amyloid protein (Aβ). Enormous SP deposition occurs in the cortices of AD patients’ brains, and this induces inflammatory responses via astroglial and microglial activation. Moreover, accumulating evidence suggests that Aβ is causative for synaptic degeneration, eventually leading to cognitive dysfunction [4]. On the other hand, NFTs are the intracellular accumulation of aggregated tau, a microtubule-binding protein [5,6,7,8]. It is widely accepted that hyperphosphorylation of tau induces its aggregation, and the severity of NFT accumulation correlates well with neuronal loss and dementia in AD patients [8]. Although mutations in certain genes cause familial AD (FAD), more than 90% of AD patients have the sporadic type, suggesting that aging is the biggest risk factor for AD. While these molecular events are closely associated with the development of AD, lifestyle choices leading to adverse medical conditions are beginning to be understood as also contributing to AD development.

The chronic consumption of foods rich in saturated fats and sugar, accompanied by physical inactivity, causes insulin resistance and obesity, leading to a variety of metabolic disorders, such as metabolic syndrome and type II diabetes mellitus (DM) [9]. These metabolic diseases greatly reduce life expectancy and are associated with elevated blood pressure, cardiovascular disease, dyslipidemia, hypercholesterolemia, and proiflammatory states [10,11]. Recently, several epidemiological and clinical studies showed that type II DM patients are more likely to develop cognitive dysfunction and exhibit increased susceptibility to AD [12,13,14,15,16,17]. Recent findings also showed that there are several similarities and connections between the pathology observed in the brains of AD and DM patients. This is especially the case for aberrant insulin signaling, supporting the idea that AD can be thought of as “type III DM” [18,19,20,21,22,23]. In the brain, insulin plays a pivotal role in neuronal functions by regulating energy metabolism, growth, survival, and differentiation via insulin signaling [24,25,26,27,28,29]. Thus aberrant insulin signaling causes an alteration in the signaling pathway, leading to an AD-like pattern of reduced cerebral glucose metabolic rate in the brain [30,31]. Histopathological evidence supports this. The experimental induction of DM enhances AD pathology, such as SP and NFT development, in several animal models [32,33,34,35,36,37,38,39,40,41,42,43].

This mini-review summarizes the histopathological evidence that DM induces AD pathology in animal models. I also discuss the idea that aberrant insulin signaling could be the key factor that induces AD pathology.

## 2. Diabetes Mellitus (DM) and β-Amyloid Protein (Aβ) Pathology

Aβ, the major component of SP, is generated from β-amyloid precursor protein (APP) through sequential cleavages by β- and γ-secretases [4,44,45]. Presenilin 1 (PS1) is the catalytic core of the γ-secretase complex, which is composed of anterior pharynx defective-1 (Aph-1), nicastrin, presenilin enhancer-2 (Pen-2), and PS1 or PS2 [46,47,48,49]. Mutations of PS1 are the predominant cause of FAD, as more than 200 mutations have been identified [50]. Some FAD-related mutations are also identified in APP itself [51]. Thus, many scientists take advantage of transgenic animal models that express FAD-related APP and/or PS1 mutants in the brain. Importantly, most FAD-related mutations selectively increase the production of longer Aβ, Aβ42, which is prone to aggregate more readily than the dominant Aβ40 species [52,53,54].

Accumulating evidence suggests that soluble Aβ oligomers induce synaptic dysfunction, an early event in AD pathology. For example, Aβ oligomers perturb axonal transport of mitochondria and vesicles containing brain-derived neurotrophic factor (BDNF) [55,56,57]. Mitochondria and BDNF play crucial roles in synaptic transmission. At presynaptic boutons, mitochondria maintain neurotransmission by producing adenosine triphosphate and by buffering synaptic calcium [58,59,60]. After secretion from the presynaptic terminal, BDNF increases spine density by interacting with postsynaptic tropomyosin receptor kinase B receptors at the target cell membrane [61,62]. Thus, impaired axonal transport of mitochondria and BDNF can cause synaptic dysfunction [63,64]. At the postsynaptic membrane, Aβ oligomers interact with glutamate receptors and dysregulate calcium influx to impair long-term potentiation and enhance long-term depression [65,66,67,68]. Aβ oligomers also alter spine morphology and decrease spine density [69,70]. These findings suggest that Aβ plays a pivotal role in AD pathogenesis, especially in synaptic degeneration. What is the connection between DM and Aβ pathology?

For DM studies, there are two established animal models. One is the streptozotocin (STZ)-induced type I DM model. STZ is a glucosamine-nitrosourea compound. After intraperitoneal injection of STZ, its metabolite has preferential cytotoxicity for β cells in pancreatic islets, resulting in insulin deficiency. Several studies show that STZ-induced type I DM aggravates Aβ pathology in the brains of APP transgenic mice, and it also increases Aβ levels, even in the brains of non-transgenic rodents [37,71,72,73,74]. Devi *et al.* showed that β-secretase levels are increased in the brains of STZ-injected mice without any changes in mRNA [72]. This finding suggests that insulin deficiency affects post-translational modification of β-secretase and enhances the β-site cleavage of APP, leading to over-generation of Aβ [72]. Intriguingly, previous studies showed that intracerebroventricular injection of STZ (icv-STZ) directly induces insulin deficiency in the brain and reproduces AD-like neurodegeneration in adult rats [75,76,77,78]. Moreover, icv-STZ also enhances Aβ pathology in the brains of transgenic mice [34,79]. For example, icv-STZ induces intracellular accumulation of Aβ oligomers accompanied by increased production of carboxy-terminal fragments from APP, the byproduct of γ-site cleavage [79]. These findings suggest that insulin deficiency could alter the activity of β- and γ-secretases to enhance Aβ production [72,79].

On the other hand, Aβ clearance is also important for regulating Aβ levels in the brain. There are several Aβ-degrading enzymes, such as neprilysin (NEP), endotherin-converting enzyme 1 (ECE-1), and insulin-degrading enzyme (IDE) [80,81,82]. In the brains of STZ-injected rats, ECE-1 levels are downregulated in both hippocampal and cortical regions, and IDE levels are also decreased in brain cortices [83]. Thus, insulin deficiency might induce Aβ pathology through a combination of increased Aβ production and decreased Aβ clearance.

Rodents on a high-fat and/or sugar diet (HFD) represent the other established animal model for DM studies, especially for type II DM [9]. HFD not only can induce insulin resistance, but also enhances Aβ pathology in several rodent models [32,42,84,85,86]. In APP transgenic mice, HFD increases the activity of γ-secretase in the brain and concomitantly decreases the activity of IDE, resulting in enhanced Aβ accumulation in the brain [85]. These findings are consistent with those observed in type I DM models [72,79,83]. Spontaneous animal models, those in which human-like disease conditions occur naturally in animals, also confirm that DM induces Aβ pathology. In both BB/Wor rat (type I DM model) and BBZDR/Wor rat (type II DM model), APP, β-secretase, and Aβ levels are all increased [87]. It is noteworthy that the type II DM model showed a much severe phenotype [87]. In another type II DM model, Otsuka Long-Evans Tokushima Fatty (OLETF) rats, Aβ levels are also increased via downregulation of NEP [88]. These findings suggest that insulin resistance can induce Aβ pathology, as well as insulin deficiency, and that aberrant insulin signaling is the likely key factor. One elegant genetic study showed that crossing an APP transgenic mouse or APP/PS1 knock-in mouse with diabetic mice (ob/ob, Nagoya-Shibata-Yasuda, or db/db) induces not only Aβ pathology but also a diabetic phenotype that shows aberrant insulin signaling [35]. This finding suggests that Aβ itself may perturb insulin signaling.

**Figure 1:**
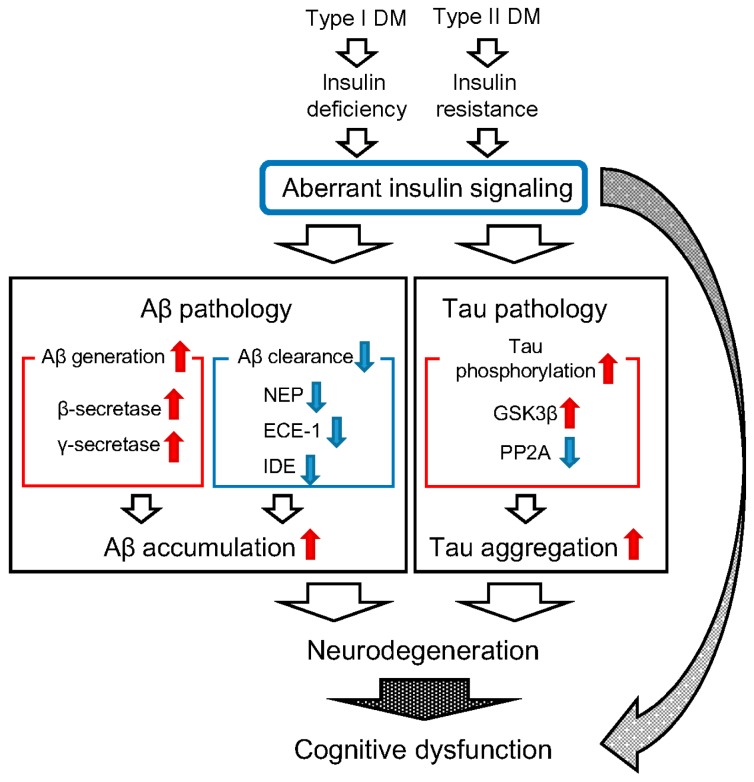
Hypothetical model illustrating the idea that diabetes mellitus induces Alzheimer’s disease (AD) pathology and cognitive dysfunction. Both type I and type II diabetes mellitus (DM) eventually cause aberrant insulin signaling. Histopathological evidence in animal model studies suggest that DM aggravates both β-amyloid protein (Aβ) and tau pathology via aberrant insulin signaling, leading to neurodegeneration. Several studies show that aberrant insulin signaling also causes cognitive dysfunction by itself. That may be why DM patients exhibit greater susceptibility toward developing AD. Modification of the insulin signaling pathway may be a promising therapeutic target for preventing cognitive dysfunction in DM and AD patients. Red arrow, increase or upregulation; blue arrow, decrease or downregulation.

Although it remains unclear why Aβ markedly accumulates in the brains of AD patients, endocytic pathology, like intraneuronal accumulation of abnormally enlarged endosomes, is frequently observed in the early stages of AD [89,90,91,92,93]. Our previous studies showed that endocytic pathology could be induced by aging alone, one that precedes SP formation in nonhuman primate brains [94,95]. Several studies show that both APP and β-secretase are transported intracellularly via endocytosis [96,97,98], and that Aβ cleavage from APP mainly occurs in endosomes [70,71]. Moreover, recent genome-wide association studies have identified AD-associated variants in endocytosis-associated genes [99,100,101,102,103]. Therefore, perturbation of endocytosis is considered to be involved in Aβ pathology. This is the line with results from our previous study demonstrating that endocytic disturbance significantly induces intracellular accumulation of Aβ [97]. Recently, we found that type II DM induces Aβ pathology in nonhuman primate brains accompanied by enhanced endocytic pathology, suggesting that type II DM aggravates age-related endocytic pathology in the brain [104]. Although additional studies are needed to clarify the precise mechanism, aberrant insulin signaling may alter intracellular endosome trafficking, leading to enhanced Aβ accumulation in the brain. Evidently, SorCS1, which is genetically associated with DM, is involved in intracellular trafficking of APP [105]. Taken together, aberrant insulin signaling might be the key factor in the induction of Aβ pathology via alteration of Aβ metabolism (Figure 1).

## 3. DM and Tau Pathology

Although FAD-related genes are associated with Aβ, the degree of dementia in AD patients and the neuronal loss correlates well with tau pathology in their brains [106]. Tau, a microtubule-associated protein, is the major component of NFTs, and hyperphosphorylation of tau is considered to be responsible for its aggregation [5,6,7,8].

One of the most relevant protein kinases involved in tau phosphorylation is glycogen synthase kinase 3β (GSK3β) [107,108,109,110,111]. Insulin and insulin-like growth factors (IGFs) mediate intracellular signaling pathways via binding to insulin receptor (IR), leading to its autophosphorylation and activation [112,113,114]. IR tyrosine kinases phosphorylate IR substrate (IRS) molecules, resulting in the activation of phosphoinositide-3 kinase (PI3K)/Akt signaling [115,116,117,118]. Importantly, the activation of the PI3K/Akt pathway results in the phosphorylation of Ser9 of GSK3β and inhibition of its kinase activity [119]. Thus, insulin deficiency/resistance can induce abnormal activation of GSK3β. Several studies show that the activity of GSK3β is enhanced in both type I and type II DM models, leading to the accumulation of hyperphosphorylated tau [30,32,34,71,120].

The activity of protein phosphatases is also important in mediating tau phosphorylation level, and the activity of certain protein phosphatases, such as protein phosphatase 2A (PP2A), is clearly decreased in AD brains [121,122,123,124]. Several studies show that DM dysregulates PP2A activity in several animal models [125,126,127]. These findings suggest that aberrant insulin signaling can alter both GSK3β and PP2A activities, leading to hyperphosphorylation of tau (Figure 1).

## 4. Conclusions

Animal model studies suggest that both type I and type II DM can aggravate AD pathology, and that aberrant insulin signaling may be the key factor (Figure 1). This is a reasonable idea, bolstered by the finding that insulin treatment ameliorates AD pathology in the brains of AD model mice [84,128]. Moreover, insulin signaling-related genes, such as *IR*, *IRS1*, *IRS2*, *IGF1*, *IGF2*, and *IGFR*, are decreased in APP/PS1 knock-in mice, suggesting that AD-related genes also affect insulin signaling by themselves [129]. Strikingly, recent findings show that a clinical trial of intranasal insulin treatment improves cognitive function of patients with AD and those in the predrome stage, mild cognitive impairment (MCI) [130,131]. Thus, modification of the insulin signaling pathway may be a promising strategy for preventing cognitive decline of patients with type II DM, MCI, and even AD.
